# Abnormality of low frequency cerebral hemodynamics oscillations in TBI population

**DOI:** 10.1016/j.brainres.2016.02.018

**Published:** 2016-03-17

**Authors:** Victor Chernomordik, Franck Amyot, Kimbra Kenney, Eric Wassermann, Ramon Diaz-Arrastia, Amir Gandjbakhche

**Affiliations:** Eunice Kennedy Shriver National Institute of Child Health and Human Development National Institutes of Health Bethesda, MD 20892, United States

**Keywords:** Cerebral autoregulation, Functional Near InfraRed, Spectroscopy, Low frequency oscillations, Parametric effect of the judgment of complexity, Traumatic brain injury

## Abstract

Functional Near Infrared Spectroscopy (fNIRS) can non-invasively capture dynamic cognitive activation and underlying physiological processes by measuring changes in oxy- and deoxy-hemoglobin levels, correlated to brain activation. It is a portable, inexpensive and user-friendly device which is easily adapted to the outpatient setting for the assessment of cognitive functions after Traumatic Brain Injury (TBI). Low frequency oscillations in hemodynamic signal, attributed in the literature to cerebral autoregulation, were assessed using recently introduced metrics, Oxygenation Variability (OV Index), obtained from oxy/deoxy-hemoglobin variations in response to mental tasks for a group of healthy control (HC, *n*=14) and TBI (*n*=29). Participants responded to an action complexity judgment task (evaluating the complexity of daily life activities by classifying the number of steps as “few” or “many”) with a varying degree of cognitive load to produce brain activation. During the task, we measured blood variations with fNIRS and analyzed OV Index changes. Mean OV indices, corresponding to high complexity tasks, are higher than that of low complexity tasks in the HC group, revealing strong parametric effect (0.039±0.017 for low, 0.057±0.036 for high, *p*-value=0.069). However, no significant difference has been recorded for the OV indexes for two different loads in the TBI group (0.055±0.033 for low, 0.054±0.035 for high, *p*=0.9). OV index metrics proves to be sensitive to chronic TBI and can potentially be used to separate subpopulations TBI vs. HC. Noticeable differences in OV index spatial distributions between subpopulations have been observed.

## 1. Introduction

The Near InfraRed (NIR) absorption spectrum of tissue is sensitive to changes in the concentration of major tissue chromophores, most importantly, hemoglobin species. Therefore, measurements of temporal variations of backscattered light can capture functionally-evoked changes of hemoglobin in the outermost cortex, and, thus, can be used to assess hemodynamic variations, induced by a specific cognitive task. Compared to other well-established brain imaging modalities, such as fMRI, this technique offers unique advantages, including superior temporal resolution (in the range of milliseconds), and spectroscopic information about temporal variations of both components of hemoglobin, (oxy-[HbO] and deoxy-[Hb]), while functional MRI can only assess deoxy-hemoglobin [Hb] changes. Additionally, NIRS instruments are much smaller/portable, cheaper, and user-friendly compared to fMRI, and can better tolerate subject motion, making study of cognitive tasks in a clinical setting both easier and less expensive.

Multi-channel functional Near InfraRed Spectroscopy (fNIRS) systems can provide 2-Dimensional (topography) maps of brain activation during cognitive, perceptual, and motor tasks. fNIRS, though inferior to fMRI in spatial resolution and depth penetration, can provide a quick, quantitative measure of local change in blood flow, in the prefrontal cortex that is resulted from a standard cognitive challenge. Properly chosen cognitive tasks recruit relevant cortical areas within individuals, allowing comparison of brain activation in subjects with neurovascular abnormalities to normative data.

Parametric designs are particularly useful in functional imaging because they provide not only an absolute level of activation in response to a stimulus, but also the derivation of a function relating a stimulus parameter to the intensity of the hemodynamic response. In previous work (Amyot et al., 2012), we adapted a cognitive activation paradigm (Krueger et al., 2009), which produces robust anterior frontal activation on fMRI, to fNIRS. We used an event-related parametric fNIRS design to investigate the intensity of brain activity among a cohort of TBI and healthy subjects who were asked to evaluate the complexity of daily life activities on the basis of normative data (Krueger et al., 2009). With fMRI, it has previously been established that activation in the medial pre-frontal cortex (PFC) correlates with the number of dichotomous decisions about the complexity of each daily life activity.

The judgment of complexity task is requiring only a dichotomous judgment. The task also activates a more anterior and medial region than the dorsolateral prefrontal cortex, which typically shows the most intense hemodynamic response to working memory tasks (Cohen et al., 1997). The usual approach to collect and analyze fNIRS data is based on continuous wave (CW) hemodynamic signals, originating from oxy- and deoxy-hemoglobin species (in healthy subjects, oxy-hemoglobin generally increases in the involved cortex area during the task, while deoxy-hemoglobin decreases) (Huppert et al., 2009).

Another method to assess cortical activation in response to cognitive tasks is through frequency analysis of cerebral hemodynamics data, as cortical hemodynamic oscillations, originating from various physiological processes (Sassaroli et al., 2012), can provide an insight into underlying neuro-vascular physiology. It should be noted that the etiology of spontaneous oscillations is not already firmly established (e.g., Schytz et al., 2010). However, low frequency oscillations (LFO) at 0.07–0.1 Hz are often linked in the literature to cerebral autoregulation (CA) (Sassaroli et al., 2012; Obrig et al., 2000; Van Beek et al., 2008), while higher frequency oscillations at 0.2–0.3 Hz result from respiration (via vascular variations and blood pooling). Spontaneous LFO, reflecting CA of cerebral vasculature, are not related to heartbeat or respiration, and their patterns are potentially a biomarker of traumatic brain injury (TBI), and traumatic vascular injury. Though detailed physiological relationship between dynamic cerebral autoregulation and low frequency oscillations (LFO) is out of the scope of this manuscript, we have hypothesized on the basis of multiple publications (see, e.g., Sassaroli et al., 2012; Obrig et al., 2000; Van Beek et al., 2008) and Schytz et al. (2010), that such link (not necessarily causal, maybe just association) exists. It should be noted that a paper of Katura et al. (2006) provides some evidence that “the origin of LFOs in cerebral hemodynamics may lie in the regulation of regional cerebral blood flow change and energetic metabolism rather than due to the systemic regulation of the cardiovascular system.”

In the current study, we performed a frequency analysis of the cerebral hemodynamic signal to investigate the link between LFO in the medial prefrontal cortex while subjects were carrying out a cognitive task, with the number of dichotomous decisions about the complexity of a daily life activity.

First, we hypothesize that the load of the performed cognitive task should be reflected in characteristics of CA in the corresponding cortical area, i.e., the more complicated the task, the higher the level of CA-related hemodynamic oscillations (LFO). To quantify this link, we introduce a novel metric, the Oxygenation Variability Index (OVI), which is able to assess these oscillatory patterns. It is based on a simplified OVI, characterizing the variability of instantaneous oxygen saturation signal in the given frequency band (accurate definition of this parameter is discussed below (Section 3)). This parameter was successfully applied in our recent analysis to follow the development of CA in children ages (Anderson et al., 2014).

The second hypothesis is that our OVI metric is sensitive enough to detect impairment in the CA, and in particular in chronic TBI subjects with traumatic microvascular injury. Traumatic microvascular injury is a frequently overlooked component of TBI. Clinical neuroimaging (CT, MRI) gives us structural information about the lesion. fMRI provides valuable functional information on the brain lesion resulting from TBI. The frontal lobe and executive function are particularly vulnerable in TBI (Hanna-Pladdy, 2007; Salazar et al., 1995; Schwab et al., 1993). It should be noted that that this region is can be easily accessed by fNIRS imaging tools, if optode arrays are applied to the hairless skin of the forehead, making the technique faster and more reliable.

## 2. Results

**Fig. 1 –:**
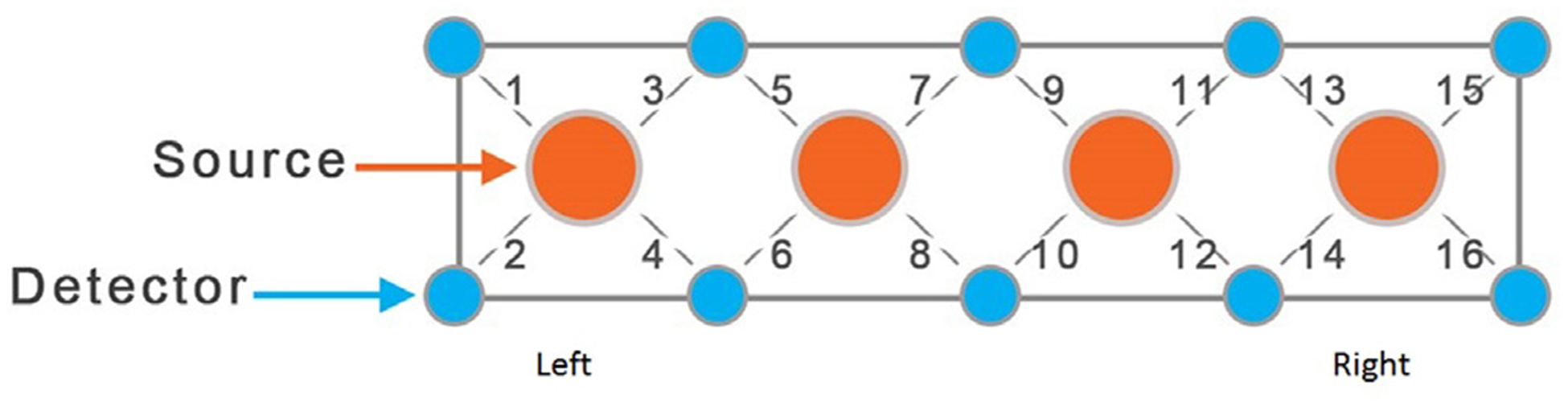
Position of fNIRS sensor, 16 channels (4 sources and 10 detectors) relative to Prefrontal Cortex during experiments.

For each patient, response times and accuracy were recorded. The HC group gives 54% of good answers and the TBI 52% with a *p*-value between the two groups of 0.39. The ratio of accuracy between the low and the high complexity task is 1.1 for the HC and 1.06 for the TBI. The *p*-value, presenting statistical significance of observed difference between the two groups is *p*=0.64. The average reaction time was 2.8 s for the HC and 2.9 s for the TBI (*p*-value=0.3). The difference in ratios between the two groups (1.1 for the HC, 1.03 for the TBI) was not statistically significant (*p*-value of 0.13) (Fig. 1).

**Fig. 2 –:**
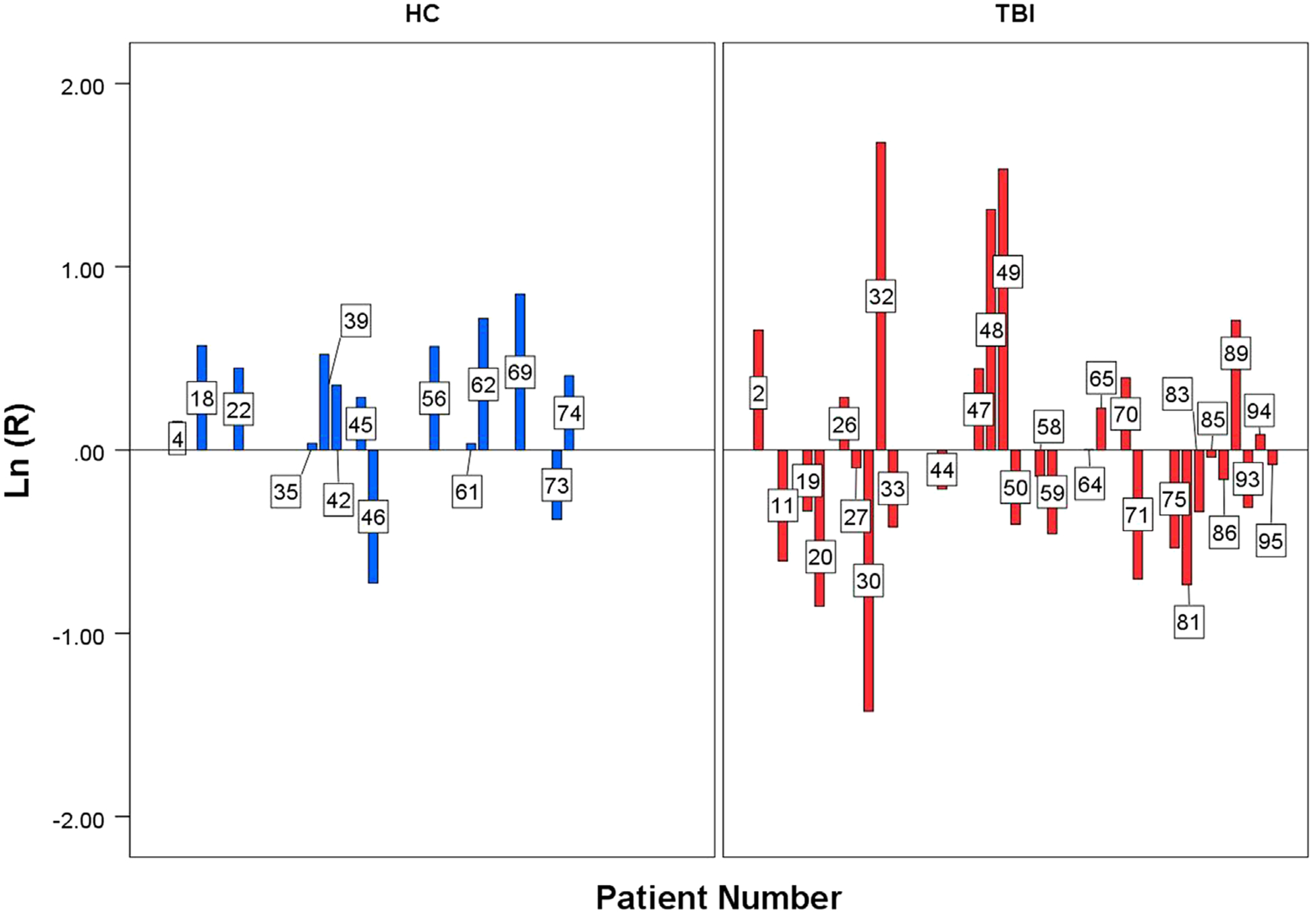
Individual subject ratio of the OV Indexes between the high and the low complexity loads, in a logarithm scale, task in 14 HC patient (blue) and 29 TBI patient (red). The ratio of each individual show an increase of OV index in the HC group between the two loads and not in the TBI group.

In Fig. 2, we show the individual subject fNIRS values *R*_*i*_ in a logarithm scale, as bar graphs for healthy control subjects (HC) and TBI (TBI), respectively. Our results demonstrate that the observed parametric effect cannot be a result of chance coincidence (for zero hypothesis that OVI responses to high and low complexity tasks are completely independent, *p*-value<^−13^): for HC subjects only 2 out of 14 subjects reveals ratio *R*_*i*_<1. In the TBI group, only 7 out of 29 have a ratio *R*_*i*_>1.

Our results show that a significant difference exists between the OV indexes for the two cognitive loads in the HC group (0.039±0.017 for low, 0.05±70.036 for high, *p*-value=0.069). However, no significant difference has been recorded for the OV indexes for two different loads in the TBI group (0.055±0.033 for low, 0.054±0.035 for high, *p*=0.9).

**Fig. 3 –:**
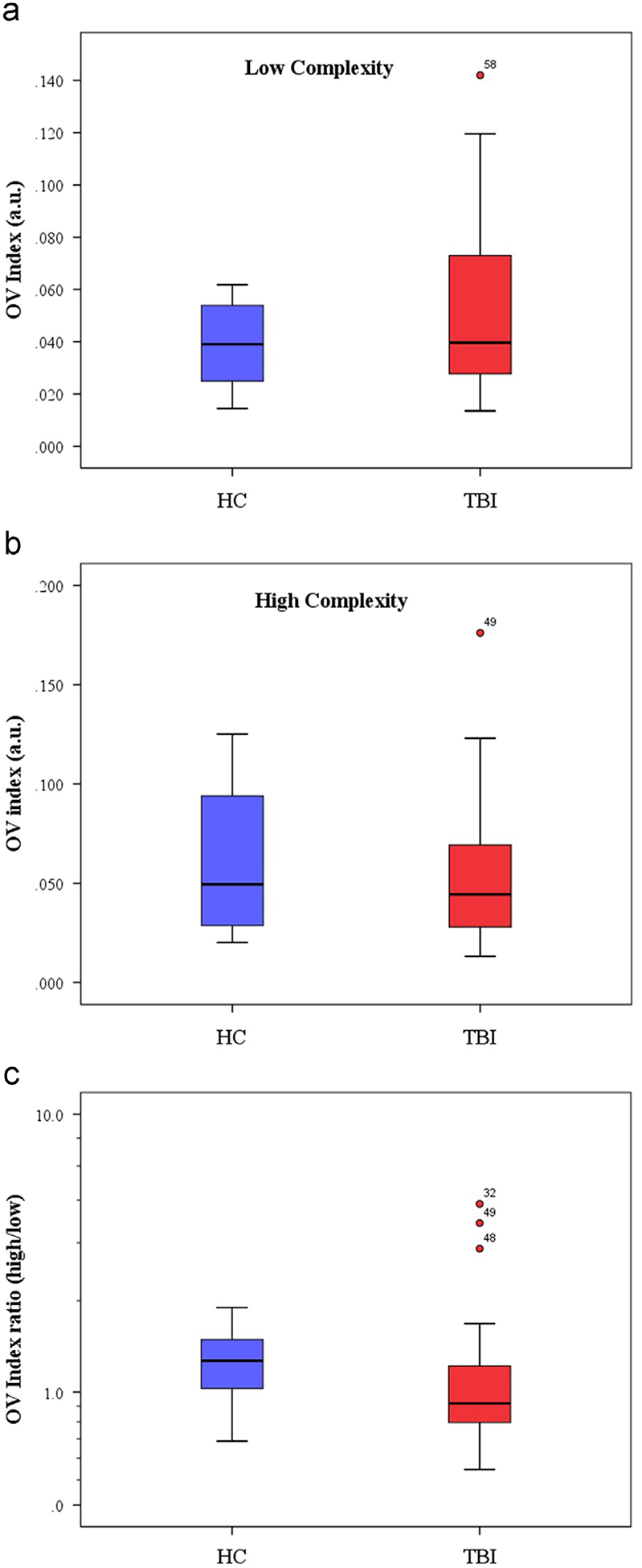
Boxplot for the low complexity task (A), high complexity (B) and the ratio of this two task in a logarithm scale (C) for Healthy control group (blue) and TBI group (red). The circles illustrate the outliers at more than one time the standard deviation and the stars illustrate the outliers at more than two times the standard deviation.

We have also measured the parametric effect of the design task by taking the ratio of the OV Indexes for the high and low complexity. Fig. 3c shows a mean ratio of 1.42±0.53 for the HC group and 1.3±1.23 for the TBI group. This difference between two groups seems to be statistically insignificant (*p*-value=0.68). However, by excluding 3 TBI outliers (i.e., patients #32, 48, 49 who have much higher OV ratios with the deviation from the mean 2 times the standard deviation of the TBI group) the *p*-value, describing the statistical significance of the difference in OV indexes for two groups becomes highly significant, corresponding to a *p*-value of 0.009. The mean ration for the TBI group without the three outliers is 0.92±0.45. We will discuss potential reason for these outliers below.

**Fig. 4 –:**
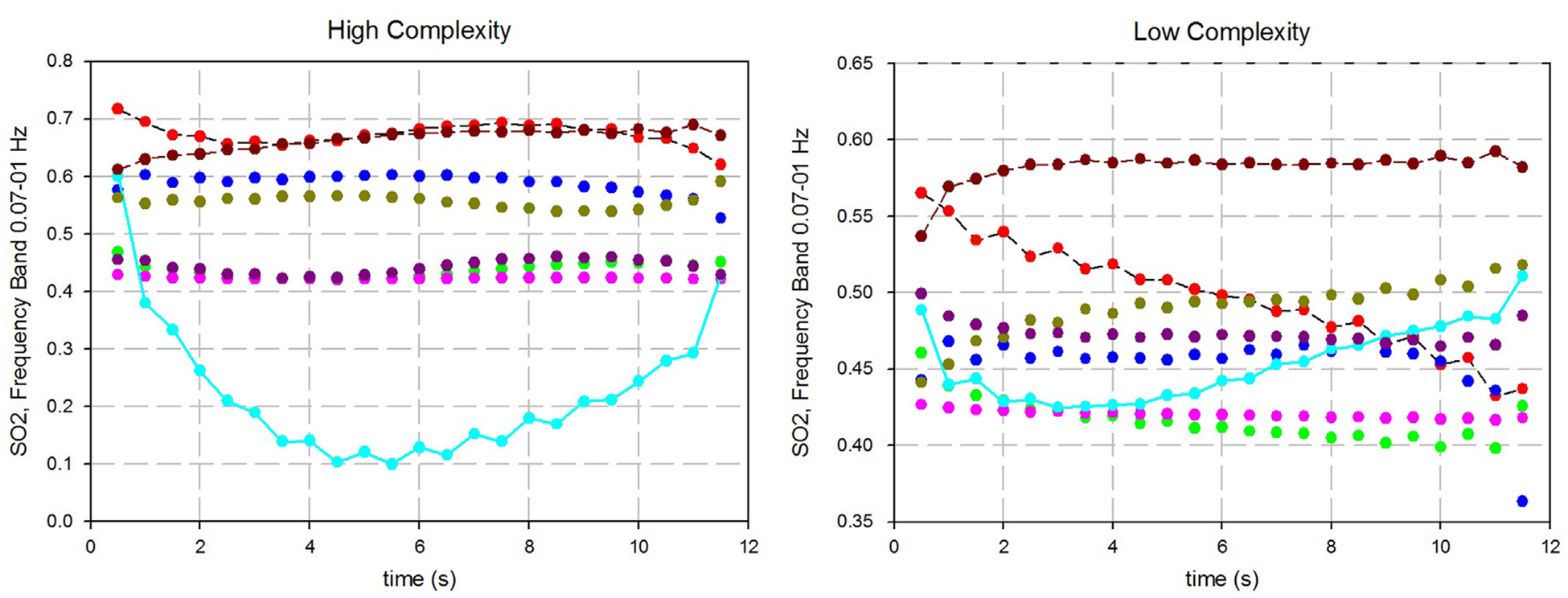
Instantaneous oxygen saturation at LFO frequency band for a healthy control subject (P. 2), as function of time after start of the mental task: low complexity task (A), high complexity (B).

We also investigated the localization of hemodynamic activation in response to a cognitive task, using OVI values in different channels as an indicator. To illustrate the fact that OVI map for healthy controls is likely to be non-uniform. We present, as an example, the pattern of instantaneous oxygen saturation SO_2_(*t*) temporal variations in different channels for subject #2 (HC) for the high and low complexity tests, respectively (Fig. 4a and b). These graphs show that response to task is localized for this healthy subject to channel 10, especially for high complexity task. SO_2_(*t*) in this channel decreases during the high complexity task (first 6 s) and then recovers during subsequent rest (next 5 s).

**Fig. 5 –:**
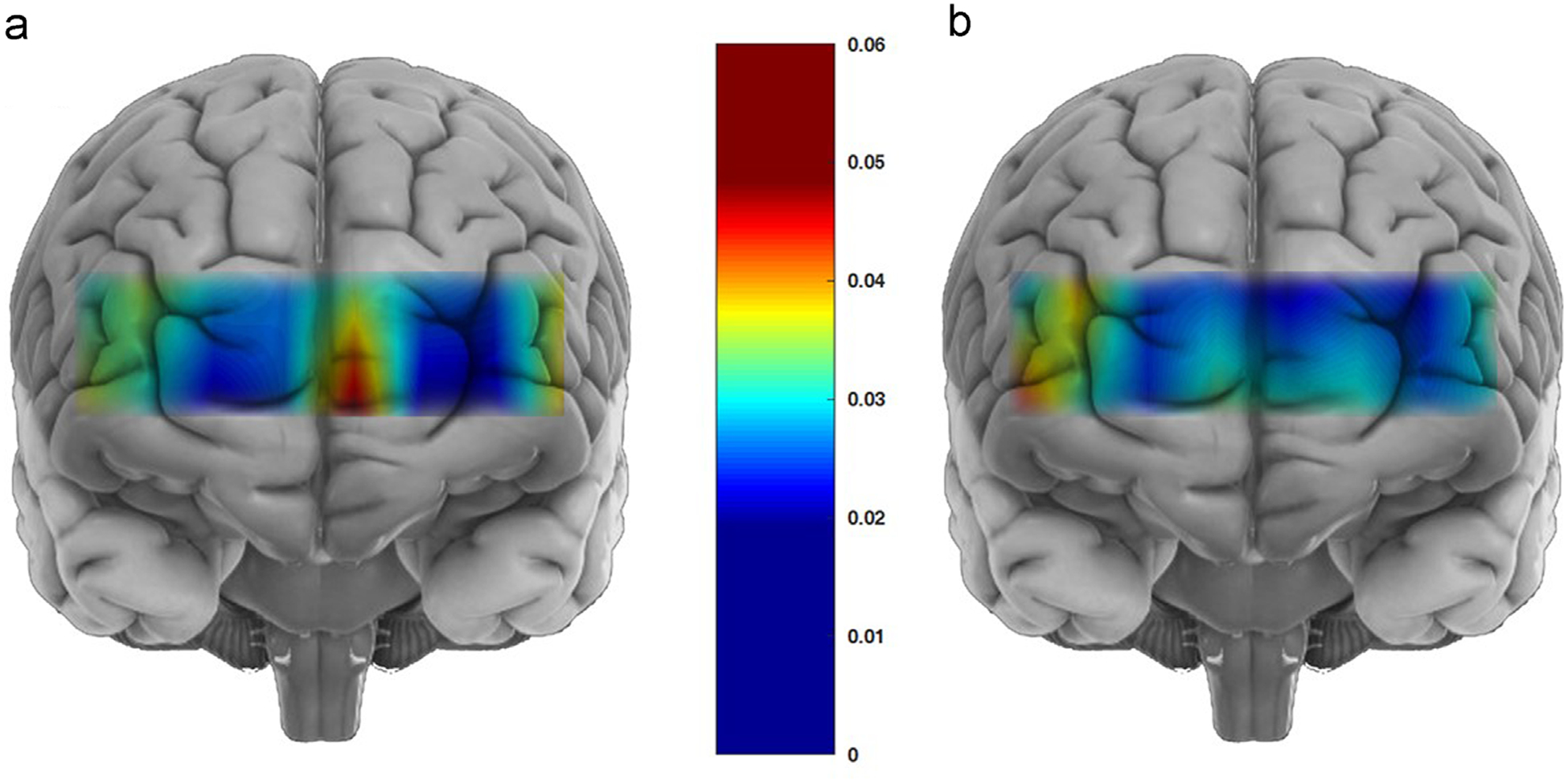
Maps of OV indexes for HC (a) and TBI (b). This map is the distribution of median values of OV index for high complexity task channel by channel, for all subjects in each of the subpopulations. The map for the healthy group shows a highly localized activation in the left prefrontal cortex, while in the TBI group, as a whole, response in cerebral autoregulation, characterized by OVI map, is broadly distributed over prefrontal cortex.

In order to better characterize the OVI localization in our cohort, we calculated the median OV indexes in each of the 16 channels, over all subjects in the group. Corresponding mapped OV indexes for the high complexity task are presented in Fig. 5a and b. These data show strong localization of OV index at channel 10 (activation area, left medial PFC) for HC subjects with but considerably dispersed distribution of the OV index over the prefrontal cortex (PFC) among TBI subjects.

## 3. Discussion and conclusions

Results of our analysis indicate that a parametric effect, i.e., differences in hemodynamic response to high and low complexity tasks, is clearly observed with the novel metrics of CA-associated OV index. Moreover, there are considerable differences in the magnitude of the parametric effect between TBI patients and healthy controls (HC). For the high complexity task, both groups perform similarly, while the significant difference between the two groups is observed in the low complexity tests. In the healthy group, there was an association between level of LFO and the complexity of the daily life activity displayed, but level of LFO was almost unchanged in the TBI group over the complexity of the daily life activity displayed. This likely indicates that TBI subjects are already operating at maximal OV index during the low complexity task.

We further investigated the specifics behind three TBI outliers in the OVI ratio metrics (Fig. 3c). From their clinical record, these 3 subjects had no cognitive impairment at the time of the medical exam. This correlates with their observed ability to modulate LFO in prefrontal cortex in accordance with complexity of the mental task, similar to healthy controls. This fact likely indicates that OVI ratio metrics, suggested here, is a promising tool to assess the degree of impairment in the TBI population, observed as reduced modulation of low frequency oscillations in response to mental tasks with different complexity levels.

The increase of LFO level between the two loads is well localized for the HC group, with maximum corresponding to channel 10. This channel (left medial prefrontal cortex) is close to the location of the activation measure, observed in fMRI or fNIRS studies in response to complexity tasks (Amyot et al., 2012; Krueger et al., 2009). Notably, among TBI subjects, this localization is lost and CA in the prefrontal cortex elicited by the difference complexity challenges is diffuse and does not change noticeably with the complexity of the task.

Our results indicate a novel metric to assess the level of cerebral hemodynamic variations using the low LFO band, which is related to cerebral autoregulation, in response to complexity cognitive tasks. This metric reveals a strong parametric effect, i.e., mean OV indexes, corresponding to high complexity tasks proved to be significantly higher than that of low complexity tasks, implying stronger CA response to more complicated tasks. Moreover, comparison between OV indexes for TBI patients and HC shows that the magnitude of OV index is sensitive to TBI and is a potential biomarker of chronic TBI, especially TBI subjects with chronic cognitive dysfunction. In our cohort, 12 of 14 healthy controls (85%) had an OVI ratio between high and low complexity tasks greater than 1, but only 4 (after excluding the same 3 outliers, previously discussed) of 26 TBI subjects (i.e., 15%) had a similar ratio, i.e., *R*>1.

In conclusion, it should be noted that the relationship between TBI and impairment in cerebral autoregulation, investigated by conventional transcranial Doppler ultrasonography, has been reported in the literature (Czosnyka et al., 2001). Novel LFO-based metrics, presented in the current paper allows to assess such impairment from a different point of view that can be potentially useful in TBI diagnostics.

## 4. Methods

### 4.1. Participants

Forty-three participants were enrolled in a USUHS IRB-approved study (NCT01789164). Participants were male (*N*=33) and female (*N*=10), aged between 18 and 60 years, and were able to read, write and speak English. The study was composed of two groups: Healthy (*N*=14) and TBI (*N*=29). TBI subject in addition should have a history of TBI with persistent concussive symptoms (according the DSM-IV research criteria for post concessional disorder) including evidence form neuropsychological testing of difficulty in attention or memory. In addition, the TBI subjects should have three or more of the following symptoms (stated shortly after the trauma and persisted for at least three months): fatigability, disorder sleep, headache, vertigo or dizziness, irritability or aggression, anxiety, change of personality, apathy or lack of spontaneity.

Both groups were matched for gender (10 males and 4 females in the HC group; 23 males and 6 females in the TBI group), age (35±3 years for HC and 37±2 years for TBI) and education (16±1 years for HC and 14±1 years for TBI).

### 4.2. Task

As mentioned above, we used an Judgment of complexity task previously shown with fMRI to produce robust activation in the anterior frontal regions (Krueger et al., 2009) and fNIRS (Amyot et al., 2012). There were two conditions: the high complexity task and the low complexity task. Stimulus presentation was controlled by the e-prime software package (Psychology Software Tools, Inc., http://www.pstnet.com/eprime.cfm). Participants were first trained on a separate set of stimuli to familiarize them with the task. At the beginning of each trial, a daily life activity task (e.g., “stirring a cup of coffee”) was displayed on a computer monitor for four seconds. Then, participants were asked to make a dichotomous decision about the complexity of the activity regarding the number of steps involved: few (e.g., “stirring a cup of coffee”) versus many (e.g., “planning a wedding”), using a two-button response pad (Krueger et al., 2009). In a previous study (Rosen et al., 2003), 114 daily life activities were rated on a Likert scale (1=few steps, 7=many steps). In this study, we presented 33 daily life activities that were rated below 3 and another 33 daily life activities with a rating above 3. Participants were told to make their decisions as quickly and accurately as possible; response times and decisions were recorded. Trials were separated by a randomly varied interval of 6–8 s. The fNIRS experiment was a single 15-min run consisting of randomly Complexity (33 low, 33 high) trials.

### 4.3. Data acquisition and analysis

In this study we used a continuous wave fNIRS system (fNIR Devices LLC, PA, USA). The instrument consists of an array of 4 sources and 10 detectors, with a total of 16 source-detector pairs (see Fig. 1). The sensor was positioned on the patient’s forehead, covering the prefrontal cortex (PFC) area of Brodmann Areas 9 and 10; sensor orientation relative to PFC is presented in Fig. 1. The system collects data at two wavelengths—730 and 850 nm—with acquisition frequency of 2 Hz. With the source-detector distance of 2.5 cm, the light is delivered to the head at a source position, and backscattered light is measured at the position of the detector. The NIR light intensities at two wavelengths are converted to changes in oxy- and deoxy-hemoglobin concentrations applying the modified Beer-Lambert law (Cope, 1991). Thus, using two wavelengths allows one to probe changes in oxy- and deoxy-hemoglobin concentrations in the cortex that are caused by brain activity, reflecting cognitive tasks.

Motion artifacts result in increased fluctuations of the CW signal during the corresponding trial. In a recent paper (Scholkmann et al., 2010) an algorithm, correcting the signal for the motion artifacts, has been proposed: 1) identifying the time intervals with strong artifacts (cw signal standard deviation σ_*0*_ is higher than chosen threshold *T* (say, *T*=0.5), 2) modifying the signal in these intervals to remove distortions. Here, we have used a simplified approach, i.e., just excluding from analysis several trials, where *σ*_0_>*T* over the trial time. It proved to be that only in one patients (#41 HC) to get more than one high complexity trial with standard deviation smaller than *T*, threshold should be increased, e.g., up to *T*=1. For reference, we present below data for this subject along with other data, but they are likely to be not reliable because of motion artifacts.

Changes in HbO and HbR concentrations were recorded for the entire experiment. For each event (33 “few” events and 33 “many” events), changes in HbO and HbR for the 11 s after stimulus presentation were extracted and averaged for each condition. Raw data on HbO and HbR changes for the 16-source/detector pairs were filtered with a narrow band elliptical filter (order 4) [0.07–0.1 Hz] and de-trended, using Matlab standard routine. Similar linear phase filters have been used in the literature for analysis of CA-related oscillations (Pierro et al., 2011).

For each channel (source/detector pair) instantaneous amplitudes *A*(*t*) and phase *φ*(*t*) of oxy- and deoxy-hemoglobin variations were obtained from the filtered NIRS data, using an algorithm, based on an analytic signal continuation approach that can be described by a formula (Boashash, 1992):

(1) $$v\left(t\right)=\text{S}\left(t\right)+jH\left|\text{S}\left(t\right)\right|=\text{A}{\left(t\right)}^{-i\varphi \left(t\right)},$$

where *S*(*t*) is the real signal, *H* denotes the Hilbert transform and *v*(*t*) indicates complex signal in the time domain. Combining the data for the two hemoglobin species (HbO and HbR) we were able to quantify instantaneous ratio of [HbO] and [Hb] signal amplitudes (as well as phase shift between the two components) in the given frequency band, as a function of time. For convenience, to characterize [HbO]/[Hb] ratio we have used directly related parameter SO_2,_ normalized to provide that its values in the range between 0 and 1, i.e.:

(2) $${\text{SO}}_{2}=\frac{\left[\text{HbO}\right]}{\left[\text{HbO}\right]+\left[\text{Hb}\right]}.$$

In a simple case of zero phase shift between oscillations of oxy- and deoxy-hemoglobin fractions, the characteristic value SO_2_, represents instantaneous oxygen saturation. However, observed variations in HbO and HbR concentrations in the chosen frequency band reflect quite complex cerebral hemodynamics: in particular, phase shifts between both components during task-rest cycle are channel dependent, revealing noticeable phase variations in just a few channels (probably related to localization of the prefrontal cortex activation). Though SO_2_ value in a given channel, as defined by Eq. (2), does not represent the oxygen saturation in the general case, this parameter can be used to characterize the ratio of [HbO] and [Hb] signal amplitudes, as a function of time and location, potentially providing information on cerebral hemodynamics, associated with response to mental tasks.

To better assess quite complicated behavior of SO_2_(*t*) during task-rest cycle in each channel and, for simplicity, we have introduced a special metrics (one number) to characterize SO_2_ level of variations during a given time interval, so-called Oxygenation Variability Index (OVI) (Anderson et al., 2014):

(3) $$OVI=\frac{\sigma \left({\text{SO}}_{2}\right)}{\mu \left({\text{SO}}_{2}\right)}$$

*OVI* is a dimensionless ratio of the standard deviation, s, and mean value, *μ*, of the evaluated SO_2_ signal, respectively.

In order to establish that the parametric effect (i.e., dependence of *OV* index on complexity of the task) is observed in obtained *OVI* data, we calculate the most robust characteristic, the ratio of mean *OV* indices for both tasks (high over low complexity) for each subject, as follows: patient #*i*, $R_{i}=\frac{<OVI_{\text{High}}^{\left(i\right)}>}{<OVI_{\text{Low}}^{i}>}$. These mean values are being calculated by averaging corresponding *OVI* channel values for the subject, e.g., $<OVI_{\text{High}}^{\left(i\right)}>\;=\;\frac{\sum_{i=1}^{N}OVI_{\text{High}}^{\left(i\right)}}{N}$, *N* ≤ 16-number of available channels.
